# The Immunologic Function of the Choroid Plexus: A Gateway to Immunomodulatory Therapy in Injury Models of the Central Nervous System

**DOI:** 10.3390/ijms27136074

**Published:** 2026-07-07

**Authors:** Roxana Rodriguez-Barrera, Yolanda Cruz-Martínez, Emilio Moreno-González, Elisa Garcia, Guadalupe Gonzalez-Pacheco, Sion Yu Jang, Adan Peña, Antonio Ibarra, Rodolfo David Mayen Quinto, Iván Ignacio Mejía, Exsal Manuel Albores-Méndez, Melchor Castro Marín

**Affiliations:** 1 Sección de Investigación, Escuela Militar de Graduados en Sanidad, UDEFA, Ciudad de Mexico 11200, Mexico; edna.garcia@anahuac.mx (E.G.); jose.ibarra@anahuac.mx (A.I.); david.invest.emgs@gmail.com (R.D.M.Q.); ivanignacio402@gmail.com (I.I.M.); albores_09@hotmail.com (E.M.A.-M.); mcmurologiahcm@hotmail.com (M.C.M.); 2 Centro de Investigación en Ciencias de la Salud (CICSA), FCS, Universidad Anáhuac Mexico Campus Norte, Huixquilucan 52786, Mexico; yolanda.cruz@anahuac.mx (Y.C.-M.); emilio.morenogo@anahuac.mx (E.M.-G.); mgonzalez.pacheco@anahuac.mx (G.G.-P.); sion_yu@anahuac.mx (S.Y.J.); adan.penab@anahuac.mx (A.P.)

**Keywords:** choroid plexus, immunomodulation, immunological function, injury model, spinal cord injury, stroke

## Abstract

Over time, our understanding of the central nervous system (CNS) as an immunologically privileged site where immune-cell infiltration takes place has changed; research has transformed the dominant view, showing that the CNS is an immunologically specialized tissue featuring complex interactions between the immune system and CNS processes, where the choroid plexus (CP) has an essential role in regulating neuronal tissue homeostasis and immune-cell trafficking. Although immune-cell entry into the CNS is tightly controlled, small numbers of antigen-experienced lymphocytes can access cerebrospinal fluid (CSF) compartments for immune surveillance under normal conditions. During an injury, such as cerebral ischemia or spinal cord damage, dendritic cell precursors infiltrate the CNS, suggesting their involvement in modulating lymphocyte activity. However, the immunoregulatory function of the CP alone is insufficient to prevent damage. Injury can trigger a cascade of events including activation of microglia toward a pro-inflammatory M1 phenotype, infiltration of peripheral immune cells across the blood–brain barrier (BBB), and uncontrolled neuroinflammation. T cells play a critical role in this process. Th1 cells exacerbate inflammation upon recognizing neural antigens, whereas Th2 cells promote recovery by releasing neurotrophic factors. This highlights the dual role of inflammation in CNS injury and repair.

## 1. Introduction

In 1885, Paul Ehrlich made a significant discovery when he administered an acid dye that stained several organs but left the brain unstained. These experiments led to the initial findings regarding the existence of the blood–brain barrier (BBB). Later, in 1908, Emil Goldmann injected various amounts of trypan blue intravenously and observed similar results, except in the choroid plexus (CP), which stained blue. He also noted that when the dye was injected directly into the cerebrospinal fluid (CSF), only the brain and spinal cord stained blue, supporting the concept of the existence of barriers which restrict the passage of substances into and out of the central nervous system (CNS). By 1929, it was established that there are several types of brain barriers with different properties, isolating the brain from the periphery and restricting the passage of molecules and cells [1].

The nature of the CNS as a nonpermissive tissue and the belief that infiltration of immune cells is a sign of pathology were firmly established. However, over the years, the research performed by Schwartz et al. and others has helped to transform this outdated notion of the immune privilege of the CNS into a realization of its role as an immunologically specialized tissue [2,3].

Three main types of BBB have been described: There is the endothelial barrier, which is made up of neurovascular units composed of an endothelial basement membrane surrounded by pericytes, astrocyte end feet, and neuronal processes, preventing passive transport from the periphery to the brain. There is the hematolicuoral barrier in the CP, which is formed by a monolayer of cuboidal epithelial cells sealed by apical tight junctions. This monolayer is separated from the capillary endothelium (unlike the BBB). It is a structure separated by the stroma of the CP, known as the Blood–Cerebrospinal Fluid Barrier (BCSFB). Finally, there is the subarachnoid barrier located in large dural and subarachnoid vessels, formed by flat arachnoid cells. The cells of the CNS barriers have unique features, including tight intercellular junctions, limited macropinocytosis, highly selective transport mechanisms, and quick responses to neuronal activity [4,5].

Recent evidence has strengthened our understanding of the communication between the CNS and the immune system. Under normal physiological conditions, T cells, macrophages, and dendritic cells can traverse the BBB and enter the brain parenchyma [6], often accessing the CNS through the BCSFB of the CP and residing in the perivascular space. Macrophages and dendritic cells serve as local antigen-presenting cells, characterized by specific cell-surface markers [7]. Activated autoreactive T cells against neuronal constituents can cross the BBB due to the expression of receptors and adhesion molecules that facilitate their migration. They undergo a clearance process in secondary lymphoid organs, which enhances their ability to enter the CNS. Inflammatory signals and cytokines further increase the permeability of the BBB, enhancing this process. Within the CNS, activated T cells interact with antigen-presenting cells (APCs). In contrast, non-activated autoreactive T cells lack these mechanisms and cannot effectively cross the BBB [8].

Following CNS injury, immune cells can exert effects through a variety of mechanisms, some antigen-dependent and others antigen-independent. The presence of antigens and molecules released from the injury site may impart either neuroprotective or neurodestructive properties to these cells. For instance, T cells can produce neurotrophic factors that enhance recovery [9]. This way of acting suggests inflammation begins with an active phase followed by one of resolution, both of which are complementary and essential for repair. Conversely, if the physiological immune response is inadequate or unresolved, it may hinder tissue regeneration and repair [3,10]. The CP stroma contains self-renewing myeloid cells, including macrophages and dendritic cells, and regulates immune-cell trafficking between the BCSFB and CSF [11]. The immunologic relevance of the CP has reemerged with current immunomodulatory therapies for ischemic CNS and spinal cord injuries; the gateway of immunological cells is the CP, and the modulation of immune cells through anti-inflammatory pathways may improve neurogenesis and provide neurological protection by containing the inflammatory process. The CP has emerged as a critical neuroimmune gateway that actively regulates communication between the CNS and the peripheral immune system. Increasing evidence indicates that CP-mediated immune responses influence the progression and resolution of neuroinflammation following cerebral ischemia and SCI. Therefore, understanding the mechanisms by which the CP controls immune-cell trafficking, antigen presentation, cytokine signaling, and CSF-mediated communication may provide novel therapeutic opportunities to improve neurological recovery. This review focuses on the immunological role of the CP in ischemic and spinal cord injury (SCI) models and discusses its potential as a target for immunomodulatory therapies.

## 2. Overview of the Plexus

### 2.1. Anatomy of the Plexuses of the Ventricles

The CP is a highly vascularized structure located within the cerebral ventricles, where it forms the BCSFB. It consists of a monolayer of cuboidal epithelial cells surrounding a connective tissue stroma that contains fenestrated capillaries, fibroblasts, and resident immune cells. The epithelial cells possess apical microvilli and are interconnected by tight junctions, which regulate the selective exchange of molecules and cells between the circulatory system and the CSF. Beyond its anatomical organization, this specialized architecture provides the structural basis for CSF production and for the CP’s role as a neuroimmune interface between the CNS and the peripheral immune system [12,13,14,15].

#### 2.1.1. Irrigation of the Choroid Plexus

The CP receives one of the richest supplies of blood in the CNS through branches of the anterior and posterior choroidal arteries. This abundant vascularization supports its high metabolic activity, continuous CSF production, and exchange of signaling molecules involved in maintaining CSF barrier homeostasis [15,16].

#### 2.1.2. The Microscopic Structure of the Choroid Plexus

The CP is composed of a highly vascularized connective tissue stroma containing fenestrated capillaries, fibroblasts, macrophages, dendritic cells, and other resident immune cells, covered by a monolayer of cuboidal epithelial cells resting on a basement membrane. Tight junctions between epithelial cells establish the BCSFB, which regulates the selective transport of molecules and immune cells between the circulatory system and the CSF. In addition to producing CSF, this specialized microarchitecture supports immune surveillance, antigen presentation, and neuroimmune communication, positioning the CP as an active interface between the CNS and the peripheral immune system [13,14,17,18,19,20].

#### 2.1.3. Central Nervous System and Lymphatic System

The fenestrated capillaries of the CP, together with hydrostatic pressure gradients, facilitate the movement of water and small molecules from the blood into the stroma; some solutes may cross via vesicular transport. Once within the stromal compartment, these solutes must traverse the choroid epithelium through specific transporters, such as those previously mentioned. Water penetrates rapidly through specialized channels, while organic substrates such as vitamins, peptides, nucleotides, and hormones are transported through active transporters. Collectively, these processes strictly regulate substrate entry and support the formation of CSF with a highly stable composition [21,22].

After CSF is produced in the CP and circulates through the ventricular system and subarachnoid space, it is reabsorbed into the venous circulation via arachnoid villi, which are projected through the dural venous sinuses. In parallel, CSF also drains via meningeal and perineural pathways, where it interfaces with local afferent lymphatic circulation, so these pathways can access the lymphatic system, thus making it possible for soluble CNS-derived antigens and immune cells to reach the nasal mucosa, where afferent lymphatics drain into the deep cervical lymph nodes (DCLNs). The presence of these lymphatic vessels in the meninges highlights an important connection between the CNS and the peripheral immune system [21].

**Figure 1:**
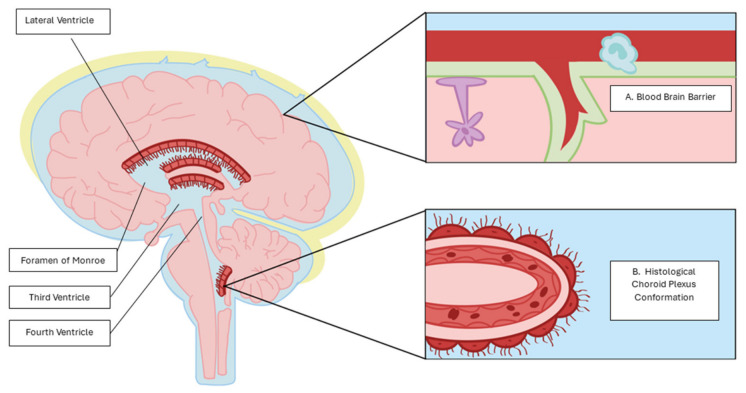
Schematic representation of the CNS barriers and lymphatic communication. The illustration depicts the BBB, the BCSFB formed by the CP epithelium, and the organization of the CP microvilli involved in cerebrospinal fluid production. The figure also illustrates the anatomical relationship between CNS barriers and lymphatic drainage pathways that contribute to immune surveillance. Prepared by the authors.

Interstitial fluid also enters the brain through the Virchow–Robin spaces. This perivascular pathway surrounds the walls of cerebral arterioles as they course from the subarachnoid space through the brain parenchyma, which is delimited characteristically by endothelial basement membrane and limitans superficial glia, in addition to perivascular glia (Figure 1) [8].

### 2.2. Choroid Plexus Function

The CP epithelium not only produces CSF but also tightly regulates its molecular composition through the coordinated activity of selective ion transporters, nutrient carriers, water channels, and efflux transport systems. This selective transport controls the entry of ions, vitamins, peptides, nucleotides, hormones, and metabolites while restricting potentially harmful blood-derived molecules, thereby maintaining CNS homeostasis. Beyond its role as a physical barrier, the CP epithelium is an active neuroimmune interface that secretes cytokines, chemokines, and growth factors into the CSF and regulates the transit of leukocytes into the CNS. During inflammation or aging, alterations in epithelial transport and secretory functions can modify CSF composition, promoting changes in immune surveillance, immune-cell recruitment, and neuroinflammatory signaling [19,22,23]. In recent years, it has been demonstrated that it also participates in memory and learning processes, regulates neurogenesis through modulation of neurogenic niche microenvironment, intervenes in the chemical surveillance of the brain [13], and has additionally been considered an immunological interface between the brain and the periphery.

In this context, the CP and other components of the brain’s chemical surveillance system play a crucial role in safeguarding the brain from toxic substances, microorganisms, and other harmful compounds. This surveillance system acts as a protective barrier, preventing the entry of potentially damaging agents into the brain. However, this system can also pose challenges in the delivery of pharmacological agents for the treatment of brain cancer, brain metastasis, neurodegenerative diseases, and brain infections. This surveillance system acts as a protective barrier, preventing the entry of potentially damaging agents into the brain. However, the CP, located within the brain ventricles, comprises a fenestrated vasculature that permits the passage of inflammatory mediators. Despite this specialized permeability, the brain’s surveillance system also poses significant challenges for the delivery of pharmacological agents used to treat brain cancer, brain metastases, neurodegenerative diseases, and brain infections [23,24,25,26].

Despite its importance, the precise mechanisms by which the components of this chemical surveillance system are regulated in response to changes in the composition of blood and brain fluids remain poorly understood. Recent studies have identified novel chemosensory receptors, such as odorant receptors, vomeronasal receptors, and taste receptors, at the brain barriers. These receptors are strategically positioned to monitor the composition of blood, CSF, and brain interstitial fluid. When these receptors bind to specific ligands, they may trigger the activity of transporters, detoxifying enzymes, or potentially other yet-unknown functions within the cells of the brain barriers. This response allows the brain to cope with alterations in the composition of blood, CSF, and interstitial fluid, effectively acting as a guardian of the CNS [22,24].

Understanding the intricate functioning of the CP and the broader chemical surveillance system is vital for developing targeted therapeutic strategies. Further research into the regulation and function of these chemosensory receptors and their interactions within the brain barriers may shed light on novel approaches to enhancing delivery of drugs to the brain while maintaining the brain’s vital protective mechanisms. Such advances could have significant implications for the treatment of a variety of neurological conditions and diseases [24].

Recent evidence further supports the notion of the CP being a metabolically active structure with sustained CSF-secretory capacity, reinforcing the relevance of CP homeostasis for CNS function. In aging rats, CP-mediated CSF secretion was shown to remain stable despite aging, supported by preserved morphology, structural integrity, gene expression, and high metabolic activity. These findings emphasize that CP function is not limited to passive barrier activity but depends on an active and highly regulated epithelial and metabolic program [26].

Beyond its role in chemical regulation and brain protection, several studies suggest that the CP is involved in neuroinflammation due to its functioning as an immunological interface between the brain and the periphery [26,27,28]. Various immune cells reside in the stroma, including macrophages, dendritic cells, natural killer (NK) cells, and T and B lymphocytes, which contribute to cerebral homeostasis and immunological surveillance [29]. Furthermore, the CP allows the controlled passage of immune cells originating from the periphery [30]. In an inflammatory context, these immune signals can modulate the choroid epithelium, reshape its secretory profile, and promote the release of chemokines and other mediators into the CSF. Additionally, studies have identified the PC as an active neuroimmunology gateway; it has been shown that T cells trafficking across the CP epithelium toward the CSF-facing (apical) side are dependent on chemokines and adhesion molecules [31]. Kolmer cells are mononuclear phagocytic cells that express β2 integrin and MHC class I and II, and their stellate morphology and close relationship with the choroid epithelium suggest an important role in regulating the passage of cells and substances through the plexuses towards the CSF [32,33]. The immune response in the CP depends on the nature of the initial pathological stimulus. In the CP, this response can modulate the immune response of brain parenchyma, probably including glial activation [34].

Under neurodegenerative, infectious, traumatic, or vascular injury conditions, the infiltration of immune cells, together with the synthesis of cytokines and inflammatory mediators in the CP, becomes highly relevant, as they can modulate the inflammatory milieu surrounding the CNS and influence processes of demyelination and neuronal death [35,36]. Under this premise, the most extensively studied models of CNS injury, such as cerebral ischemia and SCI, exhibit a pronounced neuroinflammatory response. This inflammation is driven by the recognition of neural antigens, which trigger autoreactive responses. Cerebral ischemia is characterized by cerebral blood flow reduction, leading to oxygen and nutrient deprivation, subsequent tissue damage, and neuronal death. In the context of SCI, the primary mechanical injury is followed by secondary damage, which includes inflammation, demyelination, neuronal apoptosis and necrosis, and the immune cells that recognize neural antigens. This immune response exacerbates the injury through autoreactive mechanisms. Understanding SCI involves grasping critical processes such as demyelination and neuronal death, which are exacerbated by the formation of a glial scar that restricts regeneration [36].

According to Orr et al., an SCI triggers robust secondary responses involving glial cells and inflammatory mediators, which exacerbate damage and support endogenous repair efforts [37]. The glial scar, composed of extracellular matrix proteins, serves to isolate damage, but it also inhibits neural recovery. Clifford et al. emphasize that strategies aimed at modifying the glial scar or preventing its formation are essential for improving regeneration after an SCI, with a focus on approaches such as scar prevention or resolution [38]. In this context, the CP plays a pivotal role due to its involvement in modulating neuroinflammation through CSF production. However, leveraging its therapeutic potential is impeded by the complexity of its interactions with other CNS components and the challenge of targeted intervention.

Current research strives to elucidate these complexities to optimize strategies that harness the CP’s role in SCI recovery [30,31]. Hence, animal models play a vital role in research on the involvement of the CP in conditions such as cerebral ischemia and SCI. Several models have been employed to simulate these injuries to study therapeutic interventions. Li et al. highlighted the use of rat models in tissue engineering for SCI. Ridlen et al. also discussed a range of animal models for compression SCI with a diversity of forms [39]. Moreover, Kjell and Olson also focused on rat models to understand the pathology of SCI and to test recovery strategies [40]. And finally Verstappen et al. have further contributed by evaluating and recommending a well-documented mid-thoracic rat model for SCI research models [41]. Collectively, these models provide insights into key advances in our understanding and therapeutic approaches involving the CP in neurological injuries [42].

### 2.3. Immunological Functions of the Choroid Plexus During CNS Injury

Beyond its classical role in CSF production, the CP functions as a dynamic neuroimmune interface that coordinates communication between the CNS and the peripheral immune system. Following cerebral ischemia and SCI, damage-associated molecular patterns (DAMPs), inflammatory cytokines, and chemokines released from injured neural tissue activate the CP epithelium, inducing functional changes that regulate immune-cell trafficking, antigen presentation, cytokine secretion, and inflammatory resolution [3,6,7,8,11,26,27,28,29,30,31,32].

One of the principal immunological functions of the CP is regulation of leukocyte trafficking through the BCSFB. Under physiological conditions, small numbers of antigen-experienced T cells continuously survey the CNS through this route. Following injury, increased expression of adhesion molecules and chemokines, including CCL2 and CCL19, promotes the recruitment of monocytes, macrophages, dendritic cells, and T cells into the CSF and subsequently toward injured neural tissue, where they contribute to either inflammatory damage or tissue repair depending on their activation state [3,8,11,29,32].

**Figure 2:**
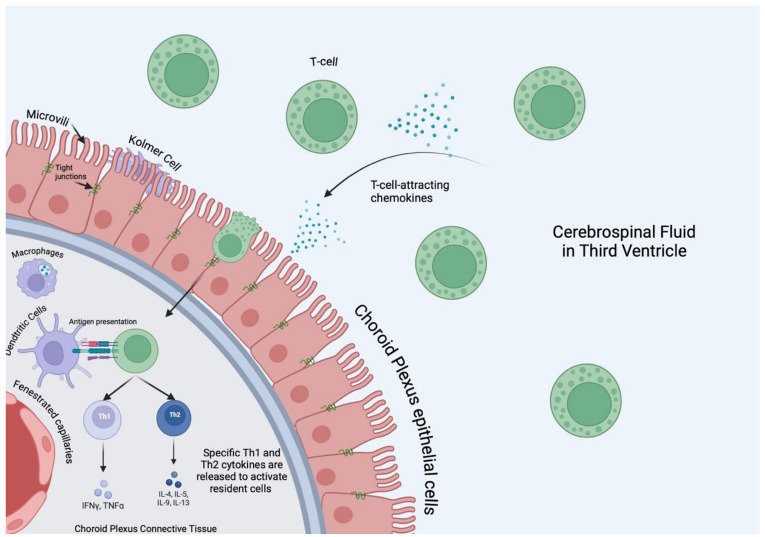
Immunological functions of the CP under physiological and inflammatory conditions. Resident macrophages, dendritic cells, and Kolmer cells participate in antigen uptake and presentation, while activated CD4+ T cells migrate across the BCSFB through chemokine- and adhesion-molecule-dependent mechanisms. Cytokines released by the CP modify the CSF microenvironment and regulate immune-cell trafficking between the peripheral immune system and the CNS. Created in BioRender. Moreno, E. (2026) https://app.biorender.com/citation/6a4561b8d01ca280024beed9 (accessed on 25 June 2026).

The CP also serves as an immunological niche for antigen presentation. Resident macrophages, dendritic cells, and Kolmer cells express major histocompatibility complex molecules and interact with infiltrating T lymphocytes, promoting their activation and differentiation according to the local cytokine milieu (Figure 2). In parallel, cytokines such as IFN-γ, TNF-α, IL-10, IL-17, and TGF-β regulate CP activity, modify its secretory profile, and influence immune-cell recruitment, neuroinflammation, and tissue repair, thereby contributing to the balance between detrimental and protective immune responses after CNS injury [3,26,27,28,29,30,31,32,33,34,35,36,37].

In addition to regulating immune-cell entry, the CP coordinates CNS-wide immune communication through the continuous production and modification of CSF. Cytokines, chemokines, growth factors, and other soluble mediators released by CP epithelial and stromal cells are distributed throughout the ventricular system and subarachnoid space, allowing inflammatory signals generated after cerebral ischemia or an SCI to influence distant CNS regions. Through this mechanism, the CP integrates local and systemic neuroimmune responses that contribute to injury progression and subsequent tissue repair [21,22,23,24,25,26,29,32].

Together, these mechanisms position the CP as an active regulator of neuroimmune responses rather than a passive barrier. By integrating immune-cell trafficking, antigen presentation, cytokine signaling, and CSF-mediated communication, the CP provides the mechanistic basis for the immunomodulatory therapeutic strategies discussed in the following sections, particularly in experimental models of cerebral ischemia and SCI.

## 3. Ischemic Injury

Cerebral ischemia is one of the most common mechanisms of acute brain injury, which causes impaired cerebral circulation that is unable to meet CNS metabolic demand. Neuron loss triggers an inflammatory cascade to clear cellular debris. The pro-inflammatory response to cerebral ischemia has been extensively studied and is known to contribute to further injury to the brain parenchyma [43]. Cerebral ischemia in humans is still a high-morbidity and -mortality disease that leads to decreased quality of life along with acute and chronic complications, hence the importance of the development of immunomodulation therapies in animal models for neuroprotection and neurogenesis [44]. Animal ischemia models are important instruments for understanding human disease, as they allow a thorough study of mechanisms and the development of new treatment options [45]. The closest animal model of ischemia with respect to human ischemic stroke is the middle cerebral artery occlusion (MCAO) model. This model is used to investigate the pathophysiology of cerebral ischemia and neurodegeneration secondary to cerebral ischemia [46].

The pathophysiology of cerebral ischemia begins with an acute phase (<24 h). The interruption of CSF deprives the brain of oxygen and glucose, leading to disrupted mitochondrial ATP synthesis and oxidative-stress-induced damage, initiated by the production of reactive oxygen species [47]. The lack of energy production creates an ionic imbalance affecting Na^+^, K^+^, and Ca^+^, which subsequently causes neuron depolarization and release of glutamate [48]. High glutamate influx into the neurons perturbs ionic homeostasis, causing Ca^2+^ overload in the mitochondria and uncoupling of the mitochondrial membrane, releasing reactive oxygen species, thereby causing cell toxicity through apoptotic pathways and cell death [49].

An important concept to consider in ischemia treatment is the ischemic core, defined as a critically hypo-perfused central area that presents irreversible damage. In contrast, the surrounding penumbra zone consists of reversibly injured tissue and is the primary target for reperfusion therapy [50]. In cases of permanent cerebral ischemia, a decrease in pH is observed, along with elevated levels of CO_2_, Ca^2+^, K^+^, and glucose, while Na^+^ levels drop. The abnormal functioning of the Na^+^ and K^+^-ATPase leads to rapid membrane depolarization, causing a massive release of glutamate, followed by a gradual decrease in its concentration. Also, the release of glutamate causes Ca^2+^-dependent channels to open, leading to a quick outflow of Ca^2+^ ions into the cortical area and a slow outflow into the basal ganglia [51,52]. Persistent cerebral ischemia induces the inflammatory process, with neutrophil infiltration occurring 12 h after the initial injury, peaking at 72 h. After 5 days of permanent ischemia, there is a greater infiltration of neutrophils and T cells, as well as activation of the microglia. Likewise, there has been evidence that monocytes and macrophages respond to DAMPs such as HMGB1 and HSPs, which activate inflammatory cells and subsequently switch to M1 and M2 phenotypes, contributing to the rupture of the BBB triggered after an ischemic stroke [52,53]. Macrophages in the subacute phase have a relevant role in phagocytizing ischemic debris, antigen processing, and presentation to lymphocytes, inducing pathway differentiation. Also, by this mechanism, nitric oxide synthase is induced to form reactive oxygen species, which oxidase lipid membranes and induce demyelination [47,48]. Furthermore, these cells produce nitric oxide synthase, which is released into the ischemic area, turning L-arginine into L-citrulline, producing nitric oxide (NO) as a metabolite. Then, NO binds to superoxide anion and produces peroxynitrite, which steals an electron from the lipid layer of the myelin and disintegrates the myelin basic protein (MBP), causing the demyelination of the adjacent brain tissue. This process is known as lipoperoxidation of the MBP [54].

The amino acid sequence MBP82-99 of MBP is commonly targeted by autoreactive T cells, which can result in demyelination and further neuronal loss of function [55]. The role of pathogenic CD4 T cell effectors has been extensively studied in experimental autoimmune encephalomyelitis (EAE) models, and IL-17 and interferon-gamma (IFN-γ) have been identified as key cytokines that confer autoimmunity. Also, the role of B cells in producing CNS-specific antigens that function as APCs for T cells has been well established [56]. However, in a model of EAE involving mice expressing the myelin oligodendrocyte glycoprotein receptor on T cells, it was discovered that depleting CD4+ T cells decreased brain B cell infiltration and lessened cognitive impairment following a stroke [57].

In contrast, in the cerebral ischemia–reperfusion model, the acid/base and electrolyte imbalance undergoes a slow reverse of pH levels, Na^+^, CO^2^, and K^+^ [51]. Two hours after reperfusion, the levels of glutamate drop drastically in the hippocampus, limiting further neuronal damage. This decrease in glutamate levels reduces the recovery of Ca^2+^ overload [45]. Reperfusion also affects immune-cell recruitment, as the extreme oxidative stress that is produced and the DAMPs released to the bloodstream lead to earlier and stronger neutrophil infiltration through the entire infarcted cortex [51]. T cells can become self-reactive, attacking brain tissues and exacerbating inflammation and damage. Phenotypes of T cells have been extensively studied, including Th1 or pro-inflammatory and anti-inflammatory Th2, because the immune response, although necessary for defense and repair, can aggravate brain damage if it is not properly regulated. Therefore, current research is looking for therapies that can modulate the immune response to promote recovery and minimize permanent damage after a stroke [57].

### 3.1. Ischemia and the Choroid Plexus 

After an ischemic stroke in the MCAO model, a sustained disruption of the ipsilateral BCSFB was observed. Notably, BCSFB alteration has also been reported contralaterally, likely driven by inflammatory cytokines within the CSF, together with changes in the SPAK: STE20/SPS1-related proline/alanine-rich kinase and Na^+^-Cl^−^ cotransporter (NCC) (SPAK-NKCC) complex and increased pro-inflammatory lipocalin (Lcn2) mRNA expression, which may contribute to CP inflammation and damage [58].

Animal model experiments have shown that the CP acts as a cell-mediated checkpoint. Inflammatory signals activate the CP epithelium, triggering immune signaling, while antigens that drain into the CSF are presented in the CP and subsequently migrate to the CNS parenchyma. This mechanism helps decrease proinflammatory subtypes of T cells and increase anti-inflammatory T cells, improving neuronal regrowth and regeneration.

**Figure 3:**
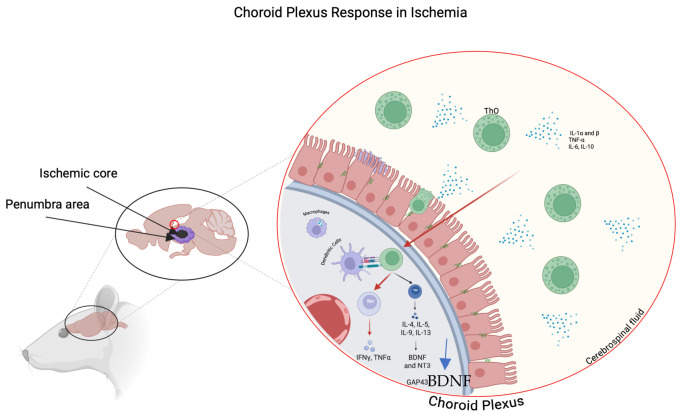
Proposed role of the CP during cerebral ischemia. Following ischemic injury, inflammatory mediators activate the CP, inducing chemokine production and recruitment of immune cells through the BCSFB. Antigen presentation within the CP promotes T-cell polarization, while cytokine secretion and neurotrophic factor production contribute to the regulation of neuroinflammation and tissue repair. Created in BioRender. Moreno, E. (2026) https://BioRender.com/8w6l3p6 (accessed on 25 June 2026).

The relevant role of the CP has previously been established as a gateway entrance for T cells in cerebral ischemia models [31]. The molecular basis for the directed migration of T cells through the CP is attributed to the presence of CC chemokine ligand 2 (CCL2), a monocyte chemoattractant protein. CCL2 is composed of a polypeptide chain with an N-terminal region that determines the affinity to its receptor and downstream signal transduction [59]. In MCAO models, several experiments have detected upregulation of CCL2 in the ischemic area 12 h after an injury and a decrease after 2 to 3 days [60]. The gradient between CCL2 expression at the site of ischemic injury and the CP induces T cells to migrate across the CP into the ischemic area [59]. The CP responds to cortical stroke by upregulating the gene expression of mediators of monocyte and T cell trafficking [61]. Furthermore, T cells residing in the CP and CSF receive specific IFN-γ and Th1 signals, prompting their differentiation into a distinct T cell subtype (Figure 3) [30].

### 3.2. Spinal Cord Injury and the Choroid Plexus

SCI is defined as temporary or permanent impairment of the spinal cord resulting in a change in its anatomy and function [62]. The most common mechanisms include high-energy injuries, infections, tumors, or vascular causes, which can be divided into traumatic and non-traumatic (reversible) injuries. SCI is a condition with high costs and rates of disability and mortality [63]. Animal models are designed to explore both physiological changes and treatment options. There are three principal injury models: compression, contusion, and transection models (incomplete or complete) [64].

The compression model emulates the flexion and axial compression that cause bone fragments to be displaced and deposited in the vertebral canal. Compression can be achieved by calibrated forceps compression, clip compression, and balloon compression. This model is suitable for understanding glial scar formation and neuroprotective therapies [65]. The contusion model is designed to simulate the crushing of the spinal vertebral column that causes the bony parts to compress the spinal cord. Injuries can be caused by a computer-controlled impactor [66]. The incomplete transection model is used when studying specific neural tracts, and different portions of the spinal cord are cut with microdissection scissors. This model can provide information about ascending and descending tract functions in neurophysiological studies [67].

The pathophysiology of SCI is divided into primary and secondary injury. Primary injury occurs due to the initial trauma, bone fragments, and ligament tearing, leading to neural parenchyma destruction, axonal communication disruption, hemorrhage, and glial membrane disruption [68]. Secondary injury is characterized by vascular damage, ionic imbalance, radial production, abnormal calcium influx, inflammation, edema, and necrosis [69]. Aspects of the pathophysiology shown in ischemia are similar; however, there are some differences, such as cell infiltration in white and gray matter, demyelination, Wallerian degeneration, and glial scar formation and M1 macrophage activation, including in the lumbar region [70].

After an SCI, immune cells do not arrive immediately at the damaged area, as the BBB is triggered by the mechanical impact, delaying the inflammatory process. However, long-lasting inflammation eventually weakens this barrier, allowing immune cells to enter the later stages. Unfortunately, the injured tissue and myelin debris promote the formation of an inflammatory microenvironment, preventing the local conversion of activated resident microglia to an M2 phenotype, attaining a resolving state. The CP has an important role in SCI; in animal models, it has been found that it functions as an orchestrator for M2 macrophage recruitment, which promotes nervous tissue repair and regeneration [3]. This event occurs because the CP releases the specific chemokines mentioned above, CCL2 and CCL19, which guide macrophages through the CP and to the injury site [69].

**Figure 4:**
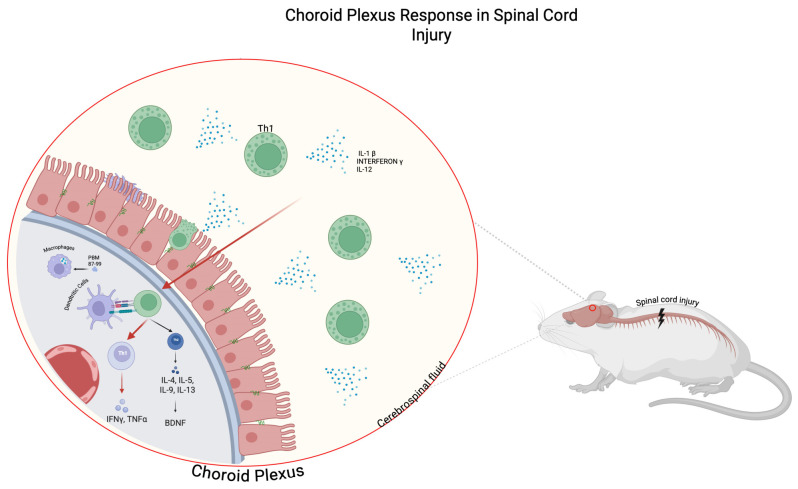
Proposed immunological role of the CP following SCI. Injury-induced inflammatory signals activate the CP, promoting chemokine secretion, antigen presentation, and immune-cell trafficking through the BCSFB. These mechanisms facilitate the recruitment of macrophages and T lymphocytes that may contribute to inflammatory regulation, tissue repair, and neurological recovery during the secondary phase of SCI. Created in BioRender. Moreno, E. (2026) https://app.biorender.com/citation/6a4557213615017f863ab87f (accessed on 25 June 2026).

Following an SCI, the CP undergoes significant physiopathological changes. As illustrated in Figure 4, antigen presentation within the CP initiates the differentiation of CD4+ T cells into Th1 or Th2 subsets, contingent on the cytokine environment present. This dynamic interaction highlights the CP’s crucial role in the immune response post-injury, as observed in a murine model [64,65].

Although the immunological role of the CP has been more extensively characterized in cerebral ischemia than in SCI, accumulating evidence indicates that similar neuroimmune mechanisms operate in both conditions. In each injury model, the CP responds to inflammatory signals by modifying its transcriptional profile, increasing chemokine expression, and regulating leukocyte trafficking across the BCSFB. However, the downstream consequences differ according to the nature of the injury. Whereas ischemia primarily involves reperfusion-induced inflammation and rapid leukocyte infiltration, SCI is characterized by a prolonged secondary injury cascade involving persistent neuroinflammation, demyelination, glial scar formation, and delayed immune-cell recruitment [3,36,37,63,64,65,66,67,68,69,70,71,72,73].

In an SCI, the CP appears to play a particularly important role in orchestrating the recruitment of repair-associated macrophages. Experimental studies have demonstrated that CP-derived chemokines, including CCL2 and CCL19, promote the migration of alternatively activated M2 macrophages toward the injured spinal cord, where they contribute to debris clearance, modulation of local inflammation, and tissue repair. In addition, resident antigen-presenting cells within the CP may influence T-cell polarization and cytokine production, suggesting that the CP contributes not only to immune-cell recruitment but also to the regulation of the inflammatory phenotype during the secondary phase of SCI [3,29,30,73].

Despite these advances, the mechanisms underlying CP-mediated immune regulation remain considerably less understood in SCI than in cerebral ischemia. Most available studies have focused on macrophage recruitment, whereas the temporal dynamics of CP activation, antigen presentation, cytokine signaling, and CSF-mediated communication after an SCI remain poorly characterized. Consequently, future studies should investigate whether therapeutic modulation of the CP can alter immune-cell trafficking and inflammatory resolution following an SCI in a manner comparable to that demonstrated in experimental models of cerebral ischemia.

To better distinguish the role of the CP in cerebral ischemia and SCI, Table 1 summarizes the main differences between both injury models, CP responses, immune mediators, therapeutic interventions, and reported outcomes.

## 4. Strategies for Therapeutics After Employing Injury Models (Ischemic and Spinal Cord Injury)

Neuroinflammation has been studied for the past twenty years, and it has been demonstrated that the triggering of the systemic inflammatory response in CNS damage may exacerbate brain and spinal injuries and perpetuate nervous tissue impairment [68,69,70,71,72,73]. Recent advances have further established the CP as a dynamic neuroimmune interface that actively regulates immune-cell trafficking, cytokine signaling, antigen presentation, and communication between the CNS and the peripheral immune system, highlighting its contribution to CNS homeostasis and the pathophysiology of neurological disorders [72]. Moreover, emerging evidence indicates that an SCI induces molecular and inflammatory alterations extending beyond the lesion’s epicenter into remote spinal segments, supporting the concept that SCI is a distributed neuroinflammatory disorder rather than an exclusively focal injury [73]. These findings reinforce the need for therapeutic strategies capable of modulating neuroimmune responses throughout the CNS. Accordingly, neuromodulation therapies have demonstrated beneficial effects by suppressing pro-inflammatory cytokines such as TNF-α and IL-12 while increasing IL-10 production, thereby promoting protective autoimmunity. In addition, myelin-specific peptide immunomodulation, including Cop-1 and A91, enhances the activation of Th2 cells, which can cross CNS barriers and secrete neurotrophic factors and anti-inflammatory cytokines such as brain-derived neurotrophic factor (BDNF), neurotrophin-3 (NT-3), IL-4, and transforming growth factor-β (TGF-β), contributing to neuroprotection and tissue repair [75].

### 4.1. Copolymer-1

Copolymer-1 or (Cop-1), also known as Copaxone, is a synthetic immunoreactive copolymer composed of four amino acids that emulate the structure of the MBP [74]. Such resemblance to the myelin structure allows the copolymer to antagonistically bind to major histocompatibility (MHC) II complexes and prevent the myelin antigens from being presented to T cells [76]. Furthermore, MHC blockage induces the differentiation of T cells into the Th2 subtypes, which exhibit an anti-inflammatory response and can move across the BBB [77]. Th2 lymphocytes induce anti-inflammatory effects by releasing cytokines such as IL-4, IL-5, IL-10, IL-13 and TGF-β that terminate immune responses and promote tissue repair and by increasing the secretion of neurotrophic factors such as insulin-like growth factor-1 (IGF-1), IGF-2 and BDNF [78].

The immunomodulatory effect of Cop-1 in the CP was studied by Cruz et al. in a model of transient ischemia in rats, in which Cop-1, along with complete Freund’s adjuvant, was administered via subcutaneous injection in the interscapular region within 5 min of reperfusion. Afterward, the CP was harvested to determine the gene expression of growth factors (BDNF, IGF-1, and NT-3) and cytokines (IL-4, IL-10, IL-17, TNF-α, and INF-γ). The study identified marked gene expression of IL-10 in the CP microenvironment while observing reduced expression of IL-17 genes. Regarding the neuronal growth factors, BDNF, NT-3, and IGF-1 underwent a significant increase in expression in Cop-1-treated rats. Subventricular and subgranular hippocampal zones were also studied to determine the neurogenesis after Cop- 1 administration; specifically, these zones were studied because they contain neural stem cells. Migration of neuroblasts from these zones was identified in Cop-1 immunized rats. The effect of Cop-1 on cerebral ischemia animal models has proven its ability to modify the infarcted brain tissue by reducing free radicals such as nitric oxide and increasing GF secretion, IL-10 secretion, IGF-1, and BDNF. This immunomodulatory effect allows the microenvironment of the site of injury to enhance the neurotrophic and neurogenic mechanisms, thus protecting the damaged tissue from further injury secondary to the pro-inflammatory response [79].

### 4.2. A91-Peptide

Another immunomodulatory peptide that has been derived is A91, a sequence 87–99 peptide obtained from the MBP, with an alanine residue instead of a lysine at position 91 [80]. Immunization with A91 peptide induces the proliferation of anti-A91 T-cells and promotes a neuroprotective effect by reducing NO and lipid peroxidation [81]. Certainly, A91 can induce Th2 subtype differentiation, promoting the secretion of BDNF, IL-4, IL-10, and TNFα. A91 peptide has been studied in SCI models, in which results demonstrated a neurogenic effect in ventral, dorsal, and ventral horns because of the anti-inflammatory effects and regeneration proteins secreted by the peptide [82]. In the ischemic zone, A91 induced anti-inflammatory gene expression, thus promoting the CP microenvironment to lean towards Th2 cell differentiation. However, A91’s immunomodulatory effect in SCI has not been studied. Theoretically, in SCI, A91 would enhance the anti-inflammatory pathways in the CP, facilitating the migration of Th2 subtype cells to the injured area, along with those affected by secondary inflammatory damage and neuron deterioration, resulting in an improved neurological prognosis. This topic should be further studied. Theoretically, in the context of SCI, A91 could enhance anti-inflammatory pathways in the CP, improving the migration of Th2 subtype cells to the site of the injury, thereby containing secondary inflammatory damage and neuronal impairment, which may improve neurological prognosis. Therefore, further studies are warranted to evaluate its potential neuroprotective effects in SCI [83].

### 4.3. Common Actions of Cop-1 and A91 Regarding the CP

Although cerebral ischemia and SCI differ in their initiating mechanisms, both injuries trigger secondary neuroinflammatory cascades in which the CP functions as a key neuroimmune interface. Through regulation of leukocyte trafficking, cytokine secretion, and immune-cell activation, the CP may influence both injury progression and tissue repair despite the distinct pathophysiological characteristics of each model. Consequently, immunomodulatory therapies targeting CP-mediated immune responses have emerged as promising experimental strategies for limiting secondary damage and promoting neurological recovery [3,31,32,38,73].

Among these agents, Cop-1 and A91 peptides have shown encouraging immunomodulatory effects in experimental CNS injury models. However, the available evidence differs considerably between these therapies. Experimental studies on cerebral ischemia have demonstrated that Cop-1 modulates the CP microenvironment by increasing the expression of IL-10 through upregulation of neurotrophic factors (BDNF and NT-3) and the growth-associated protein GAP-43 while reducing oxidative stress, thereby promoting neurogenesis and tissue repair [79,82]. These findings provide experimental evidence supporting the role of CP-mediated immunomodulation following an ischemic injury.

In contrast, A91, a modified peptide derived from myelin basic protein (MBP), has demonstrated neuroprotective effects in experimental SCI through mechanisms including reducing nitric oxide production, decreasing lipid peroxidation, and increasing expression of neurotrophic factors and anti-inflammatory cytokines [83,84]. Nevertheless, unlike Cop-1, the specific effects of A91 on CP-mediated immune regulation have not yet been directly investigated. Therefore, any proposed role of A91 in modulating immune-cell trafficking, antigen presentation, cytokine signaling, or the CP microenvironment remains hypothetical and requires direct experimental validation before comparable mechanisms can be established.

Current evidence supports the notion that the CP is a promising therapeutic target for immunomodulation following CNS injury. Experimental studies have demonstrated that the CP actively regulates immune-cell trafficking, antigen presentation, cytokine signaling, and neuroimmune communication, thereby influencing inflammatory responses and tissue repair after both cerebral ischemia and SCIs [3,11,26,27,28,29,30,31,32,38,75,81]. Nevertheless, important knowledge gaps remain regarding the molecular mechanisms through which the CP regulates immune responses in different injury models and the extent to which these experimental findings can be translated into clinical therapies. Future studies should clarify the specific contribution of CP-mediated immune regulation to neurological recovery and determine whether targeted modulation of this neuroimmune interface can improve outcomes after cerebral ischemia and SCIs [3,38,75,81]. Immunomodulatory therapies targeting CP-mediated immune responses may represent a common therapeutic strategy despite the distinct pathophysiological characteristics of each injury model.

## 5. Conclusions

Current evidence identifies the CP as a central neuroimmune interface that actively regulates communication between the CNS and the peripheral immune system. Beyond its traditional role in CSF production, the CP controls immune-cell trafficking, antigen presentation, cytokine signaling, and inflammatory responses following a CNS injury.

Experimental studies using cerebral ischemia and SCI models demonstrate that CP-mediated immune responses influence both tissue damage and repair. In ischemic injury, the CP participates in leukocyte recruitment and modulation of inflammatory signaling pathways. In SCI, the CP contributes to the recruitment of repair-associated macrophages and may support tissue regeneration through immunoregulatory mechanisms.

Immunomodulatory therapies such as Cop-1 have demonstrated the capacity to modify the CP microenvironment toward anti-inflammatory and neuroprotective responses. Although A91 peptide has shown promising neuroprotective effects in experimental models, its direct influence on CP-mediated immune regulation remains unclear and requires further investigation.

Despite substantial progress, important questions remain regarding the temporal regulation of CP responses after an injury, the molecular mechanisms governing immune-cell trafficking, and the translation of these findings into clinical therapies. Future studies focusing on CP-targeted immunomodulation may provide novel therapeutic strategies with which to enhance neurological recovery following a CNS injury.

## Figures and Tables

**Table 1:** Summary of the main differences between both injury models.

FEATURE	CEREBRAL ISCHEMIA	SPINAL CORD INJURY
Primary insult	Reduction in or interruption of CSF [42,43,44,45,46,47,48,49,50,51]	Mechanical trauma causing primary-spinal-cord damage [63,64,65,66,67,68,69,70,71]
Main secondary injury mechanisms	Excitotoxicity, oxidative stress, BBB/BCSFB disruption, reperfusion injury, leukocyte infiltration [46,47,48,49,50,51,52,53,54,55,56]	Hemorrhage, edema, demyelination, Wallerian degeneration, glial scar formation, persistent neuroinflammation [70,71,72,73]
CP response	BCSFB disruption, increased inflammatory signaling, CCL2 upregulation, trafficking of leukocytes toward ischemic tissue [29,30,57,58,59,60]	Activation of CP-mediated immune pathways, CCL2/CCL19 signaling, recruitment of repair-associated macrophages [3,29,30,73]
Main immune mediators	CCL2, IFN-γ, TNF-α, IL-17, IL-10, NO [52,53,54,55,56,57,58,59,60]	CCL2, CCL19, IL-10, TGF-β, nitric oxide [3,73,74]
Main immune cells involved	T cells, monocytes, macrophages, dendritic cells, microglia [6,7,8,9,10,11,52,53,54,55,56,57,58,59,60,73]	M2 macrophages, monocytes, T cells, dendritic cells, microglia [3,29,30,70,73]
Therapeutic interventions discussed	Cop-1 immunization after cerebral ischemia [73,74,75,76,77]	A91 peptide in experimental SCI; CP-mediated macrophage recruitment; Cop-1 as a potential immunomodulatory strategy [3,78,79,80,81,82]
Reported outcomes	Reduced oxidative stress, increase in IL-10 and neurotrophic factors, enhanced neurogenesis, reduced secondary damage [77]	Reduced NO and lipid peroxidation, increase in neurotrophic factors, macrophage-mediated repair, increase in BDNF and GAP43, improved functional recovery in experimental models [3,80,81,82]
Current evidence and limitations	CP-mediated immunomodulation has been experimentally demonstrated, although clinical translation remains limited [57,58,59,60,77]	CP participates in immune regulation after an SCI, but its molecular mechanisms remain incompletely understood; the direct effects of A91 on CP function have not yet been experimentally demonstrated [3,70,80,81,82]

## Data Availability

No new data were created or analyzed in this study. Data sharing is not applicable to this article.

## Abbreviations

The following abbreviations are used in this manuscript:

CNS	cerebral nervous system
CP	choroid plexus
CSF	cerebrospinal fluid
BBB	blood–brain barrier
BCSFB	blood–cerebrospinal fluid barrier
APCs	antigen-presenting cells
LPChA’s	choroidal arteries
mPChA’s	medial posterior choroidal arteries
ABC	ATP-binding cassette
SLC	solute carrier
NCCT and KCC1	K^+^-Cl^−^ cotransporters
NBCn1 and NCBE/NBCn2	Na^+^-HCO^3−^ cotransporters
AQP1	aquaporin-1
NHE1	Na^+^/H^+^ antiporters
NKCC1	Na^+^-K^+^-2Cl^−^ symporter
NK	natural killer
SCI	spinal cord injury
MCAO	middle cerebral artery occlusion
NO	nitric oxide
MBP	myelin basic protein
EAE	experimental autoimmune encephalomyelitis
CCL2	CC chemokine ligand 2
Cop-1	Copolymer-1
